# The complete chloroplast genome sequences of three *Pedicularis* species (Orobanchaceae)

**DOI:** 10.1590/1678-4685-GMB-2024-0010

**Published:** 2024-09-02

**Authors:** Mingcheng Wang, Shuqiao Zhang, Lei Zhang

**Affiliations:** 1 Chengdu University, Institute for Advanced Study, Chengdu, China.; 2 Engineering Research Center of Sichuan-Tibet Traditional Medicinal Plant, Chengdu, China.; 3 Chengdu University, School of Food and Biological Engineering, Chengdu, China.; 4 National Ethnic Affairs Commission, Key Laboratory of Ecological Protection of Agro-pastoral Ecotones in the Yellow River Basin, Yinchuan, China.; 5 North Minzu University, College of Biological Science & Engineering, Yinchuan, China.

**Keywords:** Hemiparasitic plants, Pedicularis, high-throughput sequencing, plastome assembly and annotation, phylogenetic analysis

## Abstract

*Pedicularis* L., a generally bothersome genus of hemiparasitic plants, is primarily native to southwestern China. The phylogenetic relationship and evolutionary history of this genus have not yet been fully resolved. In this study, we sequenced and assembled chloroplast genomes of three *Pedicularis* species, *P. chinensis*, *P. melampyriflora*, and *P. striata* using high-throughput Illumina sequencing. The assembled plastomes were 142,059 bp (*P. chinensis*) to 152,146 bp (*P. striata*) in size, containing 110 (*P. chinensis*) to 117 (*P. striata*) genes. Moreover, we identified 13-15 pseudogenes within the three plastomes, nine of which were pseudogenized in all three species. The three plastomes exhibited a similar codon usage pattern. Moreover, the plastomes contained abundant simple sequence repeats and long repeats, which showed slight variations between the three species. A maximum likelihood analysis was performed to elucidate the phylogenetic positions of the three species within the *Pedicularis* genus. The plastomes presented in our study can be used as valuable genomic resources for further genetic and genomic studies of the *Pedicularis* genus.

*Pedicularis* L., belonging to the Orobanchaceae family, is a large hemiparasitic genus containing more than 500 species that are primarily distributed in the cold mountainous regions of the Northern Hemisphere (Li et al., 2021). The majority of the *Pedicularis* species are native to southwestern China. This genus shows a high level of morphological complexity and variation, especially in the floral organs (Eaton et al., 2012). The phylogenetic relationship and evolutionary history of *Pedicularis* genus have been extensively studied in the past decade by plant taxonomists and evolutionary biologists, using high-throughput sequencing data of restriction site-associated DNA markers, nuclear and chloroplast DNA sequences, and complete chloroplast genomes (CPGs) (Eaton and Ree, 2013; Yu et al., 2013; Robart et al., 2015; Yu *et al*., 2015; Li *et al*., 2021). Among these, CPGs are powerful tools in the genomic and genetic studies of plants owing to their conservative nature and rich genetic information (Daniell et al., 2016; Wang et al., 2023). However, only 35 CPGs representing 30 *Pedicularis* species have been made available in the NCBI database thus far, which is insufficient for the comprehensive study of this genus. In this study, we sequenced the CPGs of three *Pedicularis* species, namely *P. chinensis*, *P. melampyriflora*, and *P. striata*, using high-throughput Illumina sequencing. Furthermore, we analyzed the genome structure, gene content, guanine-cytosine (GC) content, codon usage patterns, and repetitive sequences of the three CPGs. Additionally, we determined the phylogenetic positions of the three species within the *Pedicularis* genus by a maximum likelihood analysis. The well assembled and annotated CPGs presented in our study can serve as useful genomic resources for the phylogenetic, evolutionary, and genetic breeding studies of the *Pedicularis* genus.

Fresh young leaves of *P. chinensis*, *P. melampyriflora*, and *P. striata* were sampled from individual plants growing in three western provinces of China, namely Qinghai (37.1288°N, 101.7660°E), Xizang (30.8190°N, 97.3209°E), and Ningxia (35.3972°N, 106.3448°E), respectively. The voucher specimens were deposited at the Herbarium of North Minzu University (contact: Dr. Lei Zhang; zhangsanshi-0319@outlook.com) with accession number of zlnmu2022080 (*P. chinensis*), Zl20190710003 (*P. melampyriflora*), zlnmu2022098 (*P. striata*), respectively. The total genomic DNA was extracted using the cetyl trimethylammonium bromide method (Doyle and Doyle, 1987). Paired-end Illumina ReSeq libraries were constructed using an average insert size of 400 bp and sequenced on an Illumina NovaSeq 6000 platform (Illumina Inc., San Diego, CA, USA). The bcl2fastq software (Illumina) was employed to eliminate barcode sequences from the raw Illumina reads. The resulting Illumina reads were then assembled into plastomes by *de novo* assembly using NOVOPlasty (Dierckxsens et al., 2017), employing the primary parameters of “Type = chloro; Genome range = 120,000-200,000; *k*-mer = 39”, and utilizing the *P. nigra* CPG (GenBank accession number: OL544940) as both the seed input and reference sequence. The assembled CPGs were annotated using the Plann software (Huang and Cronk, 2015) with the annotation of *P. nigra* CPG as the reference. The annotated sets, encompassing protein-coding genes (PCGs), transfer RNAs (tRNAs), ribosomal RNAs (rRNAs), and pseudogenes, were manually verified. Physical circular maps of the three CPGs were generated using the web tool OGDRAW v1.3.1 (Greiner et al., 2019). The codon usage patterns of the three CPGs were analyzed by calculating the relative synonymous codon usage (RSCU) values using CodonW v1.4.2 package (Peden, 1999). Simple sequence repeats (SSRs) in the three CPGs were identified using the online MISA server (Beier et al., 2017) with default parameters. Repetitive sequences, including forward, reverse, palindrome, and complement sequences, were detected using the online REPuter server (Kurtz et al., 2001) with the hamming distance and minimal repeat size set to 3 and 30, respectively. Lastly, the CPGs of *P. chinensis*, *P. melampyriflora*, *P. striata*, and 30 other *Pedicularis* species (each represented by a single plastome) from the NCBI database were used for the phylogentic analysis, using two *Scrophularia* species (*S. dentata* and *S. henryi*) as outgroup. The coding sequences of PCGs that are present in all the 35 species were extracted from the CPGs and aligned using MAFFT-LINSI v7.313 (Katoh and Standley, 2013) with default parameters. A maximum likelihood species tree was reconstructed based on the concatenated alignments by RAxML v8.2.11 (Stamatakis, 2014) under the GTRGAMMA model with 500 replicates using the rapid bootstrap method.

We generated a total of 4.71, 6.46, and 6.71 Gb of Illumina short reads for *P. chinensis*, *P. melampyriflora*, and *P. striata*, respectively. The mean coverage depth of the Illumina reads of the three CPGs ranged between 416× to 760× (Figure S1). *De novo* assembly of these reads generated three circular CPGs (Figure 1) with genome sizes ranging from 142,059 bp (*P. chinensis*) to 152,146 bp (*P. striata*). The three CPGs have been submitted to the NCBI database with the accession numbers OQ842968 (*P. chinensis*), OQ842969 (*P. melampyriflora*), and OQ842970 (*P. striata*), respectively. The relevant information about the three CPGs is summarized in Table 1. Similar to other sequenced CPGs of the *Pedicularis* species, the three CPGs consistently showed a typical quadripartite structure, including a large single copy (LSC) region (82,437-83,466 bp), a small single copy (SSC) region (12,208-17,454 bp), and a pair of inverted repeats (IRs; 23,707-25,613 bp). Like other representative parasitic plants such as *Santalum album* (Yang et al., 2020), *Striga asiatica* (Qin et al., 2023), *Cuscuta australis* (Wang et al., 2020), and *Scurrula parasitica* (Jiang et al., 2019), the genome structures in our assembled plastomes remain highly conserved despite the degradation of photosynthetic capacities. The *P. chinensis* plastome had an overall GC content of 38.47%, which was slightly higher than that of *P. striata* (38.30%) and *P. melampyriflora* (38.33%). Among the three main regions of CPGs, the IR and SSC regions exhibited the highest (> 43%) and lowest (~32%) GC content, respectively.

**Table 1 -:** Characteristics of the plastomes of *P. chinensis*, and *P. melampyriflora*, and *P. striata*.

Species	*P. chinensis*	*P. melampyriflora*	*P. striata*
Locations	37.1288°N, 101.7660°E	30.8190°N, 97.3209°E	35.3972°N, 106.3448°E
Total sequenced bases (bp)	4,712,166,900	6,456,515,700	6,710,261,700
Coverage depth (×)	416	760	502
Genome size (bp)	142,059	151,062	152,146
LSC size (bp)	82,437	82,716	83,466
SSC size (bp)	12,208	17,128	17,454
IR size (bp)	23,707	25,609	25,613
Overall GC content (%)	38.47	38.33	38.30
GC content in LSC (%)	36.44	36.47	36.46
GC content in SSC (%)	31.53	32.42	32.33
GC content in IR (%)	43.78	43.32	43.33
Number of genes	110	112	117
Number of protein-coding genes	66	67	72
Number of tRNAs	36	37	37
Number of rRNAs	8	8	8
Number of pseudogenes	13	15	13
Number of encoded codons	18,806	18,747	21,270
Most used amino acids (Leucine)	1,900	1,940	2,188
Least used amino acids (Cysteine)	195	202	228
Codons with RSCU < 1	31	31	32
Codons with RSCU > 1	31	30	30
Codons with RSCU = 1	2	3	2
Number of SSRs	49	37	42
Number of palindromic repeats	26	29	30
Number of forward repeats	23	21	19
Number of reverse repeats	1	0	0
Number of complement repeats	0	0	1

**Figure 1 -:**
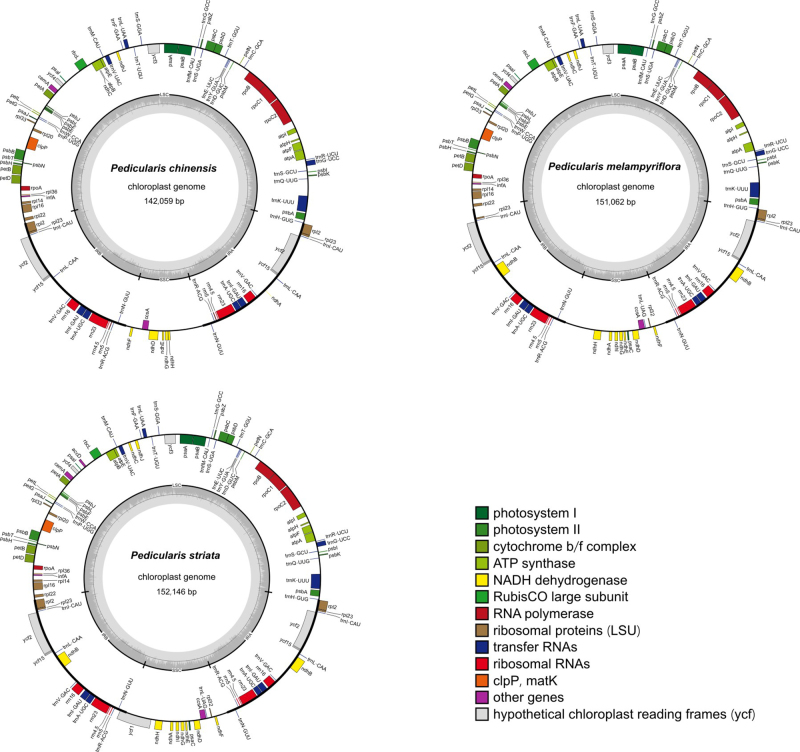
Physical circular maps of the plastomes of *P. chinensis*, *P. melampyriflora*, and *P. striata*. Genes from different functional groups are denoted in different colors. The boundaries of LSC, SSC, and IR regions are marked in the inner circle, with the dark gray bars representing the GC content.

A total of 110, 112, and 117 genes were annotated in the CPGs of *P. chinensis*, *P. melampyriflora*, and *P. striata*, respectively. The three CPGs contained similar amounts of tRNAs (36 or 37) and rRNAs (8). However, *P. striata* had more PCGs (72) compared to *P. chinensis* (66) and *P. melampyriflora* (67). A total of 58 PCGs were common among the three species, while two PCGs (*ycf1* and *accD*) were specific to *P. striata*. Four rRNAs (*rrn16*, *rrn23*, *rrn5*, and *rrn4.5*), seven tRNAs (*trnI*-*CAU*, *trnL*-*CAA*, *trnV*-*GAC*, *trnI*-*GAU*, *trnA*-*UGC*, *trnR*-*ACG*, and *trnN*-*GUU*), and three PCGs (*rpl2*, *ycf2*, and *rps7*) were found to be duplicated in the IR regions of all three CPGs. Moreover, we identified 13-15 pseudogenes within the three CPGs, nine (*ndhA*, *ndhC*, *ndhD*, *ndhE*, *ndhF*, *ndhG*, *ndhH*, *ccsA*, and *ycf15*) of which were pseudogenized in all three species (Table S1). Comparative analysis of chloroplast genomes, incorporating the three presented plastomes and six other *Pedicularis* plastomes annotated with pseudogenes, unveiled varying pseudogene counts (ranging from 2 to 15) among *Pedicularis* species, with the *ccsA* gene universally identified as pseudogenized across all examined species. The PCGs of the three CPGs encoded 18,806 (*P. chinensis*) to 21,270 (*P. striata*) codons. The three genomes exhibited a similar codon usage pattern (Figure S2A), with leucine and cysteine being the most (> 10%) and least (~1%) used amino acids, respectively. RSCU analysis demonstrated that the majority of amino acid codons exhibited significant codon usage bias, with RSCU values either > 1 (indicating higher-than-expected frequency of usage) or < 1 (indicating lower-than-expected frequency of usage) (Figure S2B), except for methionine (AUG) and tryptophan (UGG) in all three species, and serine (UCC) in *P. melampyriflora*. Furthermore, the three CPGs showed a similar distribution pattern of amino acid frequencies and RSCU values to those of *P. ishidoyana* and *S. dentata* (Figure S2), indicating a strong preservation of codon usage patterns within the *Pedicularis* genus and its associated taxa. As shown in Table 1, the *P. chinensis* CPG contained more (49) SSRs than *P. melampyriflora* (37) and *P. striata* (42). Within the three CPGs, the mono-nucleotide repeat was the most common SSR type, accounting for 48.65-52.38% of all the SSRs. Moreover, one pentanucleotide repeat was unique to *P. chinensis*, while two hexanucleotides were unique to *P. striata*. In addition, all three CPGs had 50 long repeats, including 26-30 palindromic repeats, 19-23 forward repeats, one complement repeat specific to *P. striata*, and one reverse repeat specific to *P. chinensis*. We identified a total of 52 conserved PCGs that are present in the CPGs of all 35 species. A highly supported species phylogeny was constructed, with most of the nodes having 100% rapid bootstrap support (Figure 2). In the resulting plastome phylogeny, *P. chinensis* clustered together with *P. aschistorhyncha*, *P. melampyriflora* clustered together with *P. densispica* and *P. lyrata*, while *P. striata* showed the closest genetic relationship to *P. dissecta*. Remarkably, this study presents the phylogenetic positions of *P. melampyriflora* and *P. striata* for the first time. Furthermore, the placement of *P. chinensis* differed from previous findings in studies such as Yu et al. (2015) and Robart et al. (2015), possibly due to variations in DNA sequence types and the species included for phylogeny construction.

**Figure 2 -:**
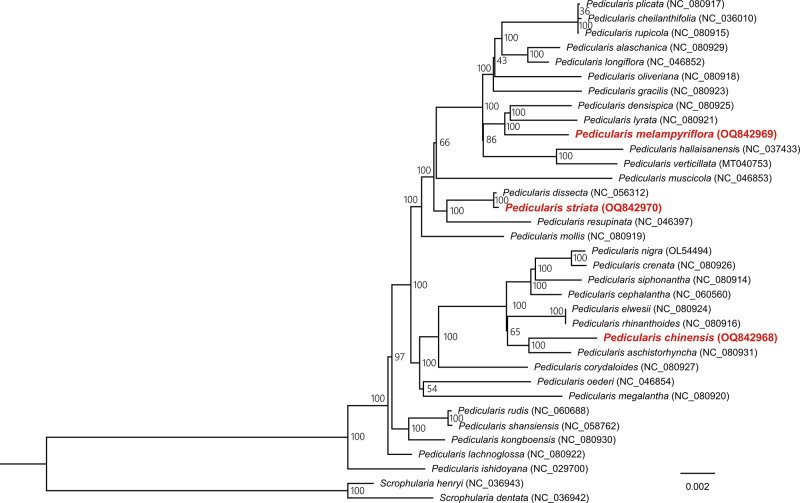
Maximum likelihood species tree of 33 *Pedicularis* species and 2 *Scrophularia* species as outgroup based on 52 conserved protein-coding genes. GenBank accession numbers were placed next to the species names. The number on each node indicates the rapid bootstrap value.

In summary, the CPGs of three distinct species dispersed at various phylogenetic positions within the *Pedicularis* genus were provided in this study. The plastomes we have disclosed can be combined with previously published *Pedicularis* plastomes to perform a comparative analysis of chloroplast genomes. This would enable us to answer a range of issues regarding genome evolution, including plastome structure evolution, gene loss or pseudogenization in hemiparasitic plants, gene selection pressure, and speciation history. Especially for *Pedicularis*, a group that is frequently debated and causes disagreement. Furthermore, the plastomes presented in this work can offer fundamental information for *Pedicularis* species identification. These plastomes can be used as valuable genomic and genetic resources for the *Pedicularis* genus and related taxa.

## Supplementary material

Figure S1 - Read depth distributions on the plastomes of *P. chinensis*, *P. melampyriflora*, and *P. striata*.

Figure S2 - Codon usage patterns in the plastomes of *P. chinensis*, *P. melampyriflora*, *P. striata, P. ishidoyana*, and *Scrophularia dentata*.

Table S1 - Pseudogenes within the plastomes of *P. chinensis*, *P. melampyriflora*, *P. striata*, and several other *Pedicularis* species.
