# Supplementary material for: The complete chloroplast genome sequences of three Pedicularis species (Orobanchaceae)
Source: Genet Mol Biol. 2024 Sep 2;47(3):e20240010. doi: 10.1590/1678-4685-GMB-2024-0010 (PMC11641048; doi:10.1590/1678-4685-GMB-2024-0010)
Supplement: Figure S1 - [file 1415-4757-GMB-47-03-e20240010-s1.pdf]

Genetics and Molecular Biology
https://doi.org/10.1590/1678-4685-GMB-2024-0010

Supplementary Material to "The complete chloroplast genome sequences of three *Pedicularis* species (Orobanchaceae)"

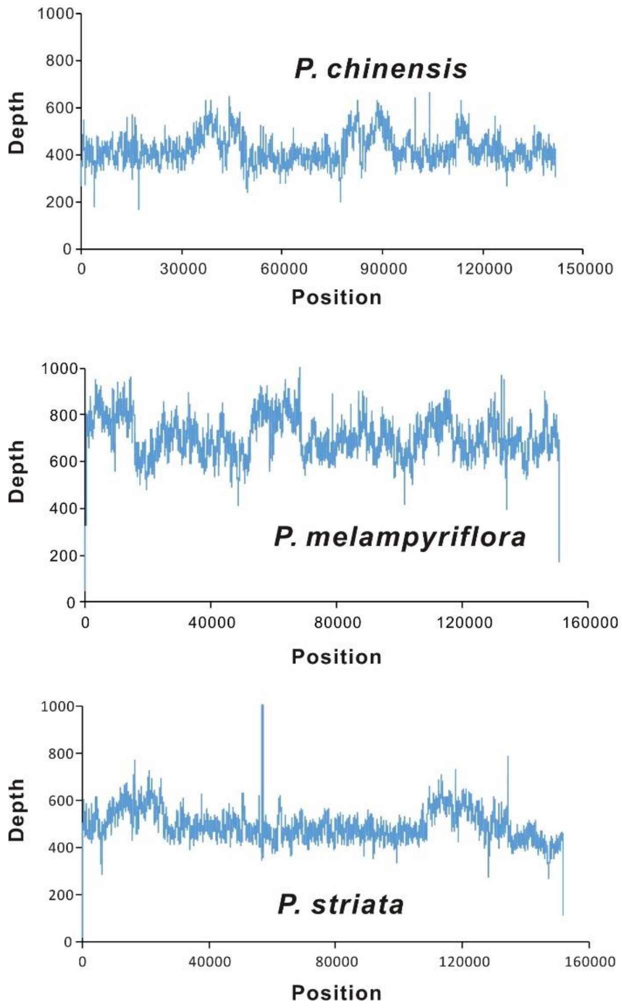

Figure S1 - Read depth distributions on the plastomes of *P. chinensis*, P. melampyriflora, and *P. striata*.
