# Supplementary material for: The complete chloroplast genome sequences of three Pedicularis species (Orobanchaceae)
Source: Genet Mol Biol. 2024 Sep 2;47(3):e20240010. doi: 10.1590/1678-4685-GMB-2024-0010 (PMC11641048; doi:10.1590/1678-4685-GMB-2024-0010)
Supplement: Table S1 - [file 1415-4757-GMB-47-03-e20240010-s3.pdf]

Genetics and Molecular Biology

https://doi.org/10.1590/1678-4685-GMB-2024-0010

Supplementary Material to "The complete chloroplast genome sequences of three *Pedicularis* species (Orobanchaceae)"

Table S1 - Pseudogenes within the plastomes of *P. chinensis*, *P. melampyriflora*, *P. striata*, and several other *Pedicularis* species. Asterisks denote the presence of two copies of pseudogenes.

**Species**	**List of pseudogenes**
P. cephalantha	*rps19*, *ccsA*
P. cheilanthifolia	*ndhC*, *rpoA*, *ndhA*, *ndhI*, *ndhE*, *ndhD*, *ccsA*, *ycf1*
P. chinensis	*ndhJ*, *ndhC*, *ycf15**, *ndhF*, *ccsA*, *ndhD*, *ndhE*, *ndhG*, *ndhH*, *ndhA*, *ndhB**
P. hallaisanensis	*ndh*, *ndhE*, *ccsA*, *ycf1*
P. melampyriflora	*ycf15**, *ndhH*, *ndhA*, *ndhI*, *ndhG*, *ndhE*, *ndhD*, *ccsA*, *ndhF*, *ndhJ*, *ndhC*, *ycf1*, *atpF*, *accD*
P. nigra	*ndhJ*, *ndhC*, *ycf15**, *ndhH*, *ndhA*, *ndhI*, *ndhG*, *ndhE*, *ndhD*, *ccsA*, *ndhF*
P. rudis	*ndhK*, *ndhD*, *ccsA*, *ndhF*
P. striata	*ycf15**, *ndhJ*, *ndhC*, *ndhH*, *ndhA*, *ndhI*, *ndhG*, *ndhE*, *ndhD*, *ccsA*, *ndhF*, *matK*
P. verticillata	*ndhJ*, *ndhK*, *ndhC*, *rpoA*, *ndhH*, *ndhE*, *ccsA*, *ycf1*
